# Voriconazole therapeutic drug monitoring and safety in HIV-infected patients with invasive fungal disease

**DOI:** 10.3389/fphar.2026.1837833

**Published:** 2026-06-19

**Authors:** Lin Hu, Yuan Su, Ju Hou, Yanfei Li, Chaogui He

**Affiliations:** 1 Department of Pharmacy, The Affiliated Changsha Hospital of Xiangya School of Medicine, Central South University, Changsha, Hunan, China; 2 Department of Pharmacy, The First Hospital of Changsha, Changsha, Hunan, China; 3 Department of Vascular Surgery, The First Hospital of Changsha, Changsha, Hunan, China

**Keywords:** HIV-infected, invasive fungal disease, safety, therapeutic drug monitoring, voriconazole

## Abstract

**Objective:**

To characterize the distribution of voriconazole (VRC) trough concentrations (*C*_trough_), identify factors associated with VRC exposure in HIV-infected patients complicated by invasive fungal disease (IFD), and determine the *C*_trough_ threshold associated with an increased risk of adverse drug reactions (ADRs).

**Methods:**

A retrospective study was conducted at the First Hospital of Changsha from 1 January 2021, to 31 December 2025. Univariate and multivariate analyses were performed to identify factors associated with VRC *C*_trough_. Receiver operating characteristic (ROC) curve analysis was used to determine the optimal cutoff value for predicting ADRs.

**Results:**

A total of 44 patients contributed 55 VRC *C*_trough_ measurements. The median *C*_trough_ was 2.44 mg/L (range, 0.07–7.26 mg/L). Of these measurements, 8 (14.5%) were <1.0 mg/L, 39 (70.9%) were within the therapeutic range (1.0–5.5 mg/L), and 8 (14.5%) were >5.5 mg/L. The interindividual coefficient of variation for *C*_trough_ was 63.6%. Univariate analysis showed that albumin-bilirubin (ALBI) grade, Child–Pugh classification, hypoalbuminemia, concomitant glucocorticoid use, C-reactive protein levels, and CD4^+^/CD8^+^ ratio were associated with VRC *C*_trough_. The mixed-effects model identified ALBI grade, concomitant glucocorticoid use, and the CD4^+^/CD8^+^ ratio as factors significantly associated with VRC *C*_trough_. The incidence of ADRs was 20.5% (9/44). Patients who developed ADRs had significantly higher median VRC *C*_trough_ than those who did not (5.15 mg/L [range, 0.88–5.93 mg/L] *vs.* 2.40 mg/L [range, 0.07–7.26 mg/L], *P* = 0.038). ROC analysis yielded an area under the curve (AUC) of 0.720 (95% confidence interval, 0.525–0.914; *P* = 0.038) for *C*_trough_ in predicting ADRs. The optimal cutoff value was 4.43 mg/L, with a sensitivity of 66.7% and a specificity of 80.4%.

**Conclusion:**

In patients with HIV-infected and IFD, VRC *C*_trough_ showed substantial variability and was influenced by multiple factors, including ALBI grade, concomitant glucocorticoid use, and the CD4^+^/CD8^+^ ratio. Maintaining VRC *C*_trough_ < 4.43 mg/L may help reduce the risk of ADRs in this population.

## Introduction

Patients with HIV-infected are highly susceptible to invasive fungal disease (IFD) because of profound immune dysfunction, and these infections remain a major cause of morbidity and mortality in this population (Dangarembizi et al., 2025; Armstrong-James et al., 2014). Voriconazole (VRC), a broad-spectrum triazole antifungal agent, is a mainstay of therapy for invasive aspergillosis and selected refractory *Candida* infections (Epelbaum et al., 2025). However, VRC displays substantial inter- and intra-individual pharmacokinetic variability, making *C*_trough_ difficult to predict reliably (Hardi et al., 2025; Coste et al., 2025).

HIV-infected patients often present with complex clinical conditions, including hypoalbuminemia, gastrointestinal dysfunction, opportunistic co-infections, and heightened inflammatory responses (Ahmed et al., 2025; Iribarren et al., 2016). These factors may influence the absorption, distribution, metabolism, and elimination of VRC, thereby further increasing variability in *C*_trough_. Consequently, fluctuations in VRC *C*_trough_ may be more pronounced in HIV-infected patients with IFD. In addition, these patients commonly receive concomitant antiretroviral therapy (ART) and may also be treated with corticosteroids, proton pump inhibitors (PPIs), and other medications during antifungal therapy. Previous studies have demonstrated significant drug–drug interactions between VRC and these agents, which may substantially alter *C*_trough_ and increase the risk of therapeutic failure or VRC-related toxicity (Arora et al., 2025; Hu et al., 2025a; Ding et al., 2026). Given the complex and multifactorial determinants of VRC pharmacokinetics in HIV-infected patients, therapeutic drug monitoring (TDM) is essential to achieve individualized dosing and optimize the efficacy and safety.

However, clinical data regarding VRC TDM in HIV-infected patients remain limited. Current prescribing information and guidelines recommend dose adjustments for patients with hepatic impairment primarily based on the Child–Pugh (CP) classification (FDA, 2025; Takesue et al., 2022). Nevertheless, emerging evidence including findings from our previous studies, has demonstrated a significant association between albumin-bilirubin (ALBI) grade and VRC *C*_trough_, suggesting that ALBI grade may serve as a useful tool for predicting VRC *C*_trough_ and hepatotoxicity (Johnson et al., 2015). Because the CP classification incorporates subjective assessments such as hepatic encephalopathy and ascites, the ALBI grade may offer a more objective and convenient alternative for estimating VRC exposure and assessing its safety.

On the basis of our previous research, the present study employed a retrospective design to collect clinical data from patients with HIV-infected who received VRC therapy with concurrent TDM. The objectives were to systematically characterize the distribution of VRC *C*_trough_ in this population, identify key factors influencing *C*_trough_, and evaluate the impact of different liver function grading systems, such as the CP class and ALBI scores on VRC exposure. Additionally, the study aimed to assess the efficacy and safety of VRC in this setting, thereby providing a scientific basis for the safe, rational, and effective use of VRC in the HIV-infected population.

## Methods

### Study design and population

This single-center retrospective study enrolled HIV-infected patients with IFD who received VRC therapy and underwent TDM at the First Hospital of Changsha between 1 January 2021, and 31 December 2025. As the Hunan Provincial Public Health Center, the institution hosts the largest HIV/AIDS care center in Hunan Province, with 100 dedicated beds and extensive expertise in HIV/AIDS management. According to the 1993 Centers for Disease Control and Prevention (CDC) criteria (NCBI, 1992), acquired immunodeficiency syndrome (AIDS) was defined as a CD4^+^ count <200 cells/µL and/or an AIDS-defining condition. The inclusion criteria were: (i) age ≥18 years; (ii) patients diagnosed with AIDS and those with confirmed HIV infection who did not yet meet the diagnostic criteria for AIDS; (iii) VRC treatment duration ≥3 days; and (iv) at least one measurement of steady-state VRC *C*_trough_. The exclusion criteria were: (i) incomplete clinical data; and (ii) concomitant use of medications contraindicated with VRC according to the prescribing information, including rifampicin, efavirenz, ritonavir, carbamazepine, and phenobarbital.

### Ethical approval

The study was approved by the Medical Ethics Committee of the First Hospital of Changsha (Approval No. 2025009). Given its retrospective design, the study did not interfere with routine clinical practice. Written informed consent was obtained from all patients enrolled in this study. All patient data were anonymized to ensure confidentiality, and the study was conducted in accordance with the principles of the Declaration of Helsinki.

### VRC administration and TDM sampling

VRC was administered according to the manufacturer’s prescribing information (FDA, 2025). Intravenous loading doses consisted of 6 mg/kg every 12 h, followed by maintenance doses of 4 mg/kg every 12 h. Oral loading doses were 400 mg every 12 h, followed by maintenance doses of 200 mg every 12 h. The route of administration and any dose adjustments were determined by the attending physicians based on individual clinical conditions. Blood samples for TDM were collected after steady-state concentrations were achieved: on day 3 of therapy for patients receiving a loading dose on day 1, and on day 4 for those who did not receive a loading dose (Chen et al., 2018). All samples were collected within 30 min prior to the next scheduled dose to determine steady-state *C*_trough_. VRC *C*_trough_ were measured using a validated liquid chromatography–tandem mass spectrometry (LC–MS/MS) method (Hu et al., 2024).

### Data collection and processing

The following data were extracted from the hospital’s electronic medical record and laboratory information systems: age, sex, body weight, date of HIV/AIDS diagnosis, most recent CD4^+^ and CD8^+^ T-lymphocyte counts, and underlying comorbidities; VRC dose, route of administration, and treatment duration; concomitant ART regimens and other potentially interacting medications, including PPIs, corticosteroids and additional antifungal agents. Laboratory and TDM data were also collected, including VRC *C*_trough_ with corresponding sampling times; liver function parameters, including alanine aminotransferase (ALT), aspartate aminotransferase (AST), and total bilirubin (TBIL); renal function indices, including serum creatinine (Scr) and blood urea nitrogen (BUN); serum albumin (ALB); and C-reactive protein (CRP) concentration. VRC-related adverse drug reactions (ADRs), efficacy, and any dose reductions or treatment discontinuations due to ADRs were recorded. The target therapeutic range for VRC *C*_trough_ was defined as 1.0–5.5 mg/L. Liver function was assessed using both the CP classification and the ALBI grade, while renal function was evaluated by creatinine clearance (Ccr). Hypoalbuminemia was defined as a serum ALB level <30 g/L (Sánchez-Rodríguez et al., 2018). CRP concentration was used as a marker for the inflammation status. Based on CRP levels, patients were categorized into three groups: mild (<40 mg/L), moderate (41–100 mg/L), and severe (>100 mg/L) inflammation (Hu et al., 2025b).

### Assessment of efficacy and safety

The diagnostic criteria for IFD, indications for VRC therapy, and evaluation of treatment efficacy were based on the updated European Organization for Research and Treatment of Cancer and the Mycoses Study Group of the National Institute of Allergy and Infectious Diseases (EORTC/MSG) (Donnelly et al., 2020). Treatment responses were categorized as follows: complete response (CR), defined as complete resolution of clinical symptoms and radiologic abnormalities attributable to IFD with negative microbiological tests; partial response (PR), defined as clinically meaningful improvement (≥50% reduction in clinical symptoms and/or radiologic findings) with negative or substantially decreased mycological markers; stable disease (SD), defined as no meaningful change (<50% improvement and <25% worsening in clinical symptoms and radiologic findings); and progressive disease (PD), defined as clinical or radiologic deterioration (≥25% increase in lesion size or the emergence of new lesions), irrespective of microbiological status. Overall effectiveness was defined as the proportion of patients achieving CR or PR at the end of VRC therapy. For patients classified as possible IFD, mycological evidence was absent and efficacy assessment relied primarily on host factors and clinical and/or radiologic criteria. Accordingly, response evaluation was based largely on changes in symptoms and imaging findings and was interpreted cautiously, with careful consideration of alternative explanations such as disease progression, co-infection with other pathogens, or noninfectious conditions. ADRs were identified through a pharmacist-led retrospective review of the electronic medical records. Two independent clinical pharmacists reviewed daily progress notes, nursing documentation, laboratory results, and concomitant medication lists. Potential ADRs were flagged using predefined clinical triggers (e.g., elevated liver enzymes, visual disturbances, hallucinations, and gastrointestinal symptoms). Causality was assessed using the Naranjo Adverse Drug Reaction Probability Scale, and only events classified as definite (score ≥9), probable (5–8), or possible (1–4) were included (Naranjo et al., 1981). Disagreements between reviewers were resolved by consensus with a third pharmacist.

### Statistical analysis

All statistical analyses were performed using SPSS software (version 25.0; IBM Corp., Armonk, NY, United States). A two-sided *P*-value <0.05 was considered statistically significant. Continuous variables with a normal distribution were expressed as mean ± standard deviation, whereas non-normally distributed variables were presented as median (range). Categorical variables were summarized as frequencies and percentages. Comparisons of categorical variables were conducted using the chi-square test or Fisher’s exact test, as appropriate. Continuous variables were compared using the Mann–Whitney U test or Kruskal–Wallis H test. Correlations between continuous variables were assessed using Pearson’s correlation coefficient. Variability in VRC *C*_trough_ was expressed as the coefficient of variation (CV = standard deviation/mean). Univariate analyses were performed to assess the associations between clinical variables and VRC *C*_trough_. Prior to multivariable analysis, multicollinearity was assessed using variance inflation factor (VIF). A linear mixed-effects model with a patient-specific random intercept was used to identify factors independently associated with VRC *C*_trough_, accounting for within-patient correlation arising from repeated measurements (55 measurements from 44 patients). Regression coefficients (β) are reported with 95% confidence intervals (CI) and two-sided *P* values. Fixed-effects *R* ^2^ metrics are not directly applicable to the mixed-effects model and are therefore not reported. Receiver operating characteristic (ROC) curve analysis was conducted to assess the predictive value of VRC *C*_trough_ for ADRs, and determine the cutoff value using the Youden index (Youden = sensitivity + specificity−1).

The ALBI score was calculated using the formula:

$$\text{ALBI}=\left(0.66\times \log_{10}\left[\text{TBIL},\mu \text{mol}/L\right]\right)\;‐\;\left(0.085\times \text{ALB }\left[g/L\right]\right)$$

ALBI grades were categorized as follows: grade 1 (≤−2.60), grade 2 (−2.59 to −1.39), and grade 3 (>−1.39), with higher scores indicating poorer liver function (Johnson et al., 2015).

## Results

### Patient characteristics

A total of 44 patients who received VRC were included in the final analysis. Of the 44 patients, 35 were diagnosed with AIDS, and the remaining nine were confirmed to be HIV-infected but had not yet met the diagnostic criteria for AIDS. The cohort was predominantly male (38/44, 86.4%), with a median age of 48.5 years (range, 18–77 years) and a median body weight of 53 kg (range, 38–90 kg). Regarding the duration of HIV infection, 28 patients (63.6%) had been diagnosed for less than 1 year, whereas 16 (36.4%) had a disease duration of 1 year or longer. IFD was classified as proven in 18 patients (40.9%), probable in 5 (11.4%), and possible in 21 (47.7%). VRC was administered as targeted therapy in 23 patients (52.3%) and as empirical therapy in 21 (47.7%). Most patients had pulmonary IFD (42/44, 95.5%). Fungal culture results were available for 18 patients; *Aspergillus spp.* Were isolated in 16 cases and *Candida spp.* In 2. Twelve patients (27.3%) required intensive care unit (ICU) admission, and 24 (54.5%) had hypoalbuminemia. The median CRP concentration was 31.50 mg/L (range, 0.40–254.00 mg/L). Patient characteristics are summarized in Table 1.

**TABLE 1:** Patient characteristics (*n* = 44).

Parameters	Value
Male: Female, n (%)	38 (86.4): 6 (13.6)
Age (years), median [range]	48.5 (18–77)
Body weight (kg), median [range]	53 (38–90)
Duration of HIV infection, n (%)	
<1 year	28 (63.6)
>1 year	16 (36.4)
IFD diagnosis, n (%)	
Proven	18 (40.9)
Probable	5 (11.4)
Possible	21 (47.7)
Treatment indication, n (%)	
Therapeutic	23 (52.3)
Empirical	21 (47.7)
Sites of IFD	
lung	42 (95.5)
Intestine	1 (2.3)
Central nervous system	1 (2.3)
Fungus cultured	
*Aspergillus*	16 (36.4)
*Candida*	2 (4.5)
Patients in the ICU, n (%)	
Yes	12 (27.3)
No	32 (72.7)
Duration of VRC administration (d), median [range]	15.5 (4–57)
Administration routes, n (%)	
Intravenous	30 (68.2)
Oral	14 (31.8)
Concomitant medications, n (%)	
PPIs^a^	22 (50.0)
Glucocorticoids^b^	11 (25.0)
Antiretroviral drugs^c^	39 (88.6)
CD4^+^ T lymphocytes/(cells/μl), median [range]	55 (1–738)
CD8^+^ T lymphocytes/(cells/μl), median [range]	318 (53–2,245)
CD4^+^/CD8^+^, median [range]	0.18 (0.01–1.54)
Opportunistic infections	
Pneumocystis jirovecii pneumonia	12 (27.3)
Cytomegalovirus infection	9 (20.5)
Tuberculosis	9 (20.5)
Mycobacterial infection	8 (18.2)
Talaromycosis	6 (13.6)
Nontuberculous mycobacterial infection	4 (9.1)
Cryptococcal infection	3 (6.8)
Herpes simplex virus infection	1 (2.3)
Hypoalbuminemia, n (%)	
Yes	24 (54.5)
No	20 (45.5)
ALBI grade, n (%)	
Grade 1	5 (11.4)
Grade 2	31 (70.5)
Grade 3	8 (18.2)
CP class, n (%)	
CP-A	28 (63.6)
CP-B	13 (29.5)
CP-C	3 (6.8)
Ccr (mL/min), median [range]	95.45 (8.73–324.14)
CRP (mg/L), median [range]	31.50 (0.40–254.00)
<40 mg/L	26 (59.1)
41–100 mg/L	10 (22.7)
>100 mg/L	8 (18.2)

IFD, invasive fungal disease; ICU, intensive care unit; VRC, voriconazole. *C*_trough_, trough concentration. PPIs, proton pump inhibitors; ALBI, albumin-bilirubin; CP, Child-Pugh. ccr, creatinine clearance rate. CRP, C-reactive protein.

^a^ The PPIs, used were rabeprazole (*n* = 11), pantoprazole (*n* = 9) and esomeprazole (*n* = 2).

^b^ The glucocorticoids administered were dexamethasone (n = 6; 5 mg/day in five patients and 2.5 mg/day in one patient), methylprednisolone (n = 4; 40 mg/day in one patient and 20 mg/day in three patients), and hydrocortisone (n = 1; 300 mg/day).

^c^ The antiretroviral regimens administered included lamivudine/dolutegravir (n = 19), bictegravir/emtricitabine/tenofovir alafenamide (n = 12), bictegravir/emtricitabine/tenofovir alafenamide + albuvirtide (n = 5), lamivudine/dolutegravir + albuvirtide (n = 2), darunavir/cobicistat + albuvirtide (n = 1).

### VRC use and variability of *C*_trough_

A total of 55 VRC *C*_trough_ measurements were included in the final analysis. The median number of VRC *C*_trough_ measurements per patient was 1 (range, 1–4). The median *C*_trough_ was 2.44 mg/L (range, 0.07–7.26 mg/L). Of these measurements, 8 (14.5%) were <1.0 mg/L, 39 (70.9%) were within the therapeutic range (1.0–5.5 mg/L), and 8 (14.5%) > 5.5 mg/L. The median duration of VRC therapy was 15.5 days (range, 4–57 days). VRC was administered primarily intravenously (30/44, 68.2%), while 14 patients (31.8%) received oral therapy. Only five patients (11.4%) received a loading dose. Concomitant medications included ART in 39 patients (88.6%), PPIs in 22 (50.0%), and glucocorticoids in 11 (25.0%). ART regimens included lamivudine/dolutegravir (n = 19), bictegravir/emtricitabine/tenofovir alafenamide (n = 12), bictegravir/emtricitabine/tenofovir alafenamide + albuvirtide (n = 5), lamivudine/dolutegravir + albuvirtide (n = 2), darunavir/cobicistat + albuvirtide (n = 1). The specific ART regimens and their corresponding VRC *C*_trough_ values are shown in Table 2. VRC *C*_trough_ was not significantly correlated with the administered dose (*P* = 0.605). The interindividual CV for the initial VRC *C*_trough_ was 63.6%.

**TABLE 2:** Specific ART regimens with corresponding VRC *C*_trough_ values.

Patient ID	ART regimens	VRC *C*_trough_ values
1	Bictegravir/Emtricitabine/Tenofovir Alafenamide + Albuvirtide	2.44
2	Bictegravir/Emtricitabine/Tenofovir Alafenamide + Albuvirtide	0.88
3	Bictegravir/Emtricitabine/Tenofovir Alafenamide	4.84
4	Bictegravir/Emtricitabine/Tenofovir Alafenamide	3.07
5	Lamivudine/Dolutegravir + Albuvirtide	2.43
6	NA	5.20
7	Bictegravir/Emtricitabine/Tenofovir Alafenamide + Albuvirtide	5.43
8	NA	2.36
9	Lamivudine/Dolutegravir	3.57
10	Lamivudine/Dolutegravir	1.54
11	Lamivudine/Dolutegravir	5.38
12	Lamivudine/Dolutegravir	1.56
13	Bictegravir/Emtricitabine/Tenofovir Alafenamide	5.63
14	Lamivudine/Dolutegravir	5.86
15	Bictegravir/Emtricitabine/Tenofovir Alafenamide	5.96
16	Bictegravir/Emtricitabine/Tenofovir Alafenamide	3.15
16	Bictegravir/Emtricitabine/Tenofovir Alafenamide	3.73
17	Lamivudine/Dolutegravir	2.41
17	Lamivudine/Dolutegravir	1.07
18	Lamivudine/Dolutegravir	3.63
19	Bictegravir/Emtricitabine/Tenofovir Alafenamide	3.85
20	Lamivudine/Dolutegravir	0.07
20	Lamivudine/Dolutegravir	2.39
21	NA	5.79
21	NA	4.25
22	NA	4.42
23	Lamivudine/Dolutegravir	2.03
24	Lamivudine/Dolutegravir	0.20
25	Lamivudine/Dolutegravir	0.28
25	Lamivudine/Dolutegravir	1.52
25	Lamivudine/Dolutegravir	1.02
25	Lamivudine/Dolutegravir	1.57
26	Bictegravir/Emtricitabine/Tenofovir Alafenamide	3.19
27	Bictegravir/Emtricitabine/Tenofovir Alafenamide	5.15
28	Bictegravir/Emtricitabine/Tenofovir Alafenamide + Albuvirtide	0.14
28	Bictegravir/Emtricitabine/Tenofovir Alafenamide + Albuvirtide	1.85
29	Lamivudine/Dolutegravir	3.09
30	Lamivudine/Dolutegravir	0.42
30	Lamivudine/Dolutegravir	1.67
31	Bictegravir/Emtricitabine/Tenofovir Alafenamide	5.69
32	Bictegravir/Emtricitabine/Tenofovir Alafenamide	2.34
33	Bictegravir/Emtricitabine/Tenofovir Alafenamide + Albuvirtide	5.93
33	Bictegravir/Emtricitabine/Tenofovir Alafenamide + Albuvirtide	5.47
34	Lamivudine/Dolutegravir + Albuvirtide	0.321
35	Bictegravir/Emtricitabine/Tenofovir Alafenamide	5.88
36	Darunavir/Cobicistat + Albuvirtide	1.93
37	Bictegravir/Emtricitabine/Tenofovir Alafenamide	7.26
37	Bictegravir/Emtricitabine/Tenofovir Alafenamide	1.04
38	Lamivudine/Dolutegravir	0.89
39	Lamivudine/Dolutegravir	4.44
40	Lamivudine/Dolutegravir	3.50
41	Lamivudine/Dolutegravir	1.18
42	Lamivudine/Dolutegravir	2.68
43	Lamivudine/Dolutegravir	1.66
44	NA	1.43

### Univariate analysis of factors influencing VRC *c*_trough_

**FIGURE 1:**
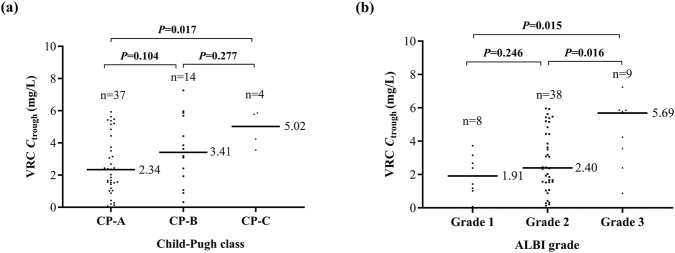
Correlation of Child–Pugh class **(a)** and ALBI grade **(b)** with VRC C_trough_. In each panel, the horizontal line indicates the median value. The *P* value and the number of C_trough_ measurements are shown above each plot. VRC, voriconazole. C_trough_, trough concentration. CP, Child–Pugh. ALBI, albumin-bilirubin.

A significant positive correlation was observed between VRC *C*_trough_ and ALBI score (*P* = 0.001). Patients with ALBI grade 3 had a higher median VRC *C*_trough_ (5.69 mg/L; range, 0.89–7.26 mg/L) compared with those with ALBI grade 1 (1.91 mg/L; range, 0.07–3.73 mg/L) and grade 2 (2.40 mg/L; range, 0.14–5.96 mg/L) (*P* = 0.015 and *P* = 0.016, respectively). No significant difference was observed between ALBI grades 1 and 2 (*P* = 0.246). The median VRC *C*_trough_ in CP-C patients was 5.02 mg/L (range, 3.57–5.88 mg/L), which was significantly higher than that observed in CP-A patients (2.34 mg/L; range, 0.07–5.93 mg/L; *P* = 0.017). No significant differences in VRC *C*_trough_ were found between CP-A and CP-B patients or between CP-B and CP-C patients (*P* = 0.104 and *P* = 0.277, respectively). The relationships among CP class, ALBI grade, and VRC *C*_trough_ are illustrated in Figure 1.

**FIGURE 2:**
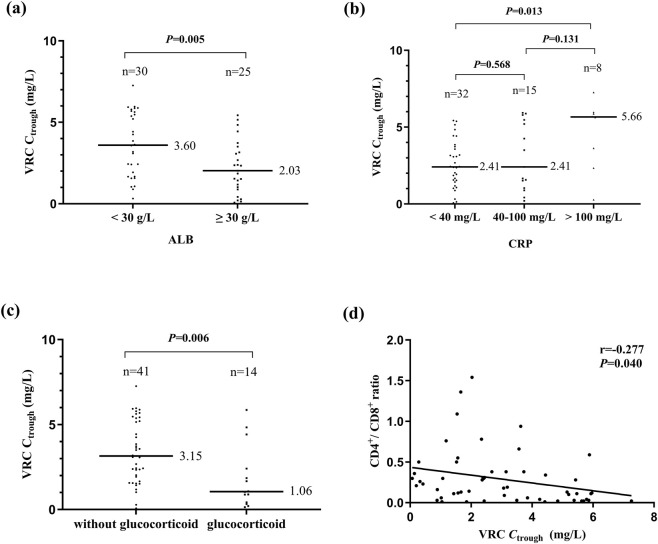
Correlation of ALB **(a)**, CRP **(b)**, concomitant glucocorticoid use **(c)**, and CD4^+^/CD8^+^ ratio **(d)** with VRC *C*_trough_. In each panel, the horizontal line indicates the median value. The *P* value and the number of *C*_trough_ measurements are shown above each plot. VRC, voriconazole. *C*_trough_, trough concentration. ALB, albumin. CRP, C-reactive protein.

Hypoalbuminemia was present in 54.5% of patients, and VRC *C*_trough_ were significantly higher in patients with hypoalbuminemia compared with those without (*P* = 0.005). Patients receiving concomitant glucocorticoid therapy had significantly lower VRC *C*_trough_ than those not receiving glucocorticoids (*P* = 0.006). In addition, VRC *C*_trough_ was positively correlated with CRP concentration (*P* = 0.007), and patients with CRP levels >100 mg/L showed a significantly increased VRC *C*_trough_ than those with CRP levels <40 mg/L (*P* = 0.013). VRC *C*_trough_ was weakly but significantly negatively correlated with the CD4^+^/CD8^+^ ratio (r = −0.277, *P* = 0.040), indicating that lower CD4^+^/CD8^+^ ratios were associated with higher VRC exposure. A sensitivity analysis stratified by CD4^+^ count (<200 cells/µL *vs.* ≥ 200 cells/µL) was conducted; this stratification revealed no statistically significant difference in VRC *C*_trough_ (*P* = 0.883). No other factors exhibited a significant correlation with VRC *C*_trough_ in the univariate analysis (*P* > 0.05). The relationships between VRC *C*_trough_ and hypoalbuminemia, CRP group, concomitant glucocorticoid use, and CD4^+^/CD8^+^ ratio are illustrated in Figure 2.

### Multivariate analysis of factors influencing VRC *c*_trough_

Six variables were significant in univariate analysis: ALBI grade, CP class, ALB, glucocorticoid use, CRP, and CD4^+^/CD8^+^ ratio. VIF analysis showed elevated values for ALBI grade (up to 8.78) and ALB (4.68), indicating collinearity among liver function markers (all other VIF <2). Because ALBI grade, ALB, and CP class reflect overlapping dimensions of hepatic function, simultaneous inclusion of all three variables may introduce multicollinearity, reducing precision and attenuating their individual effects. We therefore retained ALBI grade and excluded ALB and CP class from the final model. Therefore, only ALBI grade was retained from the liver function variables. The final mixed-effects model included ALBI grade, glucocorticoid use, CRP, and CD4^+^/CD8^+^ ratio. As shown in Table 3, ALBI grade was significantly associated with VRC *C*_trough_. Compared with ALBI grade 1, grade two showed a positive but non-significant association (β = 1.19, 95% CI: −0.04 to 2.42, *P* = 0.057), while grade 3 was significantly associated with higher VRC *C*_trough_ (β = 2.50, 95% CI: 0.77 to 4.22, *P* = 0.005). Concomitant use of glucocorticoids was significantly associated with lower VRC *C*_trough_ (β = −2.34, 95% CI: −3.34 to −1.33, *P* < 0.001). Additionally, a higher CD4^+^/CD8^+^ ratio was significantly associated with lower VRC *C*_trough_ (β = −1.93, 95% CI: −3.23 to −0.63, *P* = 0.004). CRP concentration showed a positive trend but did not reach statistical significance (β = 0.01, 95% CI: 0 to 0.02, *P* = 0.063).

**TABLE 3:** Linear mixed model for the factors affecting the VRC *C*_trough_.

Variable	β (95%CI)	*P* Value
ALBI grade^a^		
Grade 2	1.19 (−0.04, 2.42)	.057
Grade 3	2.50 (0.77, 4.22)	0.005
Combined use of glucocorticoids^b^	−2.34 (−3.34, −1.33)	<0.001
CD4^+^/CD8^+^	−1.93 (−3.23, −0.63)	0.004
CRP	0.01 (0, 0.02)	0.063

The estimated random-intercept variance was 2.189, and the intraclass correlation coefficient (ICC) was 0.668.

^a^ Compared to ALBI, grade 1.

^b^ Compared with patients not receiving concomitant glucocorticoids.

### Efficacy and safety assessment

The antifungal therapy achieved an overall effectiveness rate of 77.3% (34/44). A sensitivity analysis restricted to patients with proven or probable IFD (n = 23) showed an efficacy rate of 78.3% (18/23), comparable to the 77.3% observed in the overall cohort. This finding suggests that inclusion of patients with possible IFD did not materially bias the efficacy assessment. ADRs occurred in nine patients (20.5%), including three cases of visual disturbances, three cases of hallucinations, one case of abnormal liver function, one case of elevated Scr, and one case of gastrointestinal symptoms. The median time to onset of ADRs was 6 days (range, 3–11 days). Four patients discontinued VRC therapy due to ADRs. Patients who developed ADRs exhibited significantly higher median VRC *C*_trough_ compared with those who did not (5.15 mg/L [range, 0.88–5.93 mg/L] *vs.* 2.40 mg/L [range, 0.07–7.26 mg/L], *P* = 0.038). The individual VRC *C*_trough_ values for the nine patients who developed ADRs are presented in Table 4.

**TABLE 4:** The individual VRC *C*_trough_ values for the nine ADR cases.

Patient ID	VRC *C*_trough_ values
1	0.88
2	2.43
3	5.63
4	5.79
5	2.03
6	5.15
7	5.93
8	5.88
9	4.44

### ROC curve analysis for ADRs prediction

**FIGURE 3:**
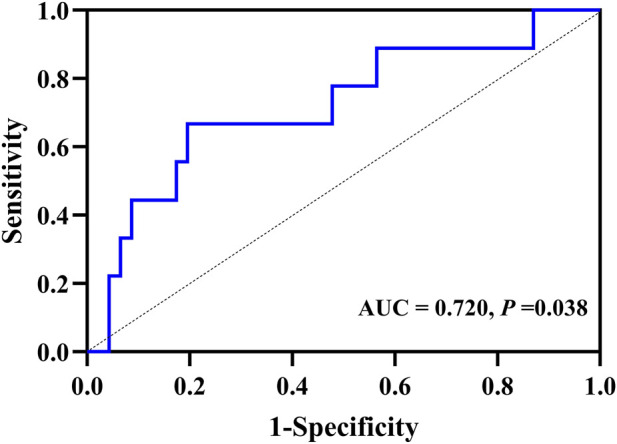
Receiver operating characteristic (ROC) curve of VRC *C*_trough_ to predict risk of ADRs. AUC, area under the curve. VRC, voriconazole. *C*_trough_, trough concentration. ADRs, adverse drug reactions.

The ROC analysis included 55 VRC *C*_trough_ measurements, of which 9 were from patients who developed ADRs and 46 were from those who did not. The predictive value of VRC *C*_trough_ for ADRs was evaluated using ROC curve analysis. As shown in Figure 3, the area under the ROC curve (AUC) for *C*_trough_ in predicting ADRs was 0.720 (95% confidence interval: 0.525–0.914; *P* = 0.038). The optimal cutoff value, determined by the maximum Youden index, was 4.43 mg/L. At this threshold, the sensitivity and specificity for predicting ADRs were 66.7% and 80.4%, respectively, suggesting that this concentration may serve as a clinically meaningful threshold for identifying patients at increased risk of ADRs. The positive predictive value (PPV) was 40.0%, and the negative predictive value (NPV) was 92.5%. In a sensitivity analysis excluding two patients with mild ADRs, the optimal cutoff was 4.28 mg/L (AUC 0.735), consistent with the primary analysis. In addition, we conducted a sensitivity analysis excluding all “possible” ADRs (n = 3). The results were consistent, with an optimal cutoff of 4.28 mg/L and an AUC of 0.731.

## Discussion

This study examined a clinically complex and high-risk population, patients with HIV-infected complicated by IFD, and systematically assessed the distribution patterns and influencing factors of VRC *C*_trough_. The findings revealed marked interindividual variability in VRC *C*_trough_, with a CV of 63.6%, and 29% of measurements had concentrations outside the therapeutic range. These results underscore the importance of TDM in HIV-infected patients and point to the increased risk of treatment failure or toxicity with standard dosing regimens in this population. Overall, this study offers valuable insights to support individualized antifungal therapy in this vulnerable group.

A core finding of this study was a significant positive correlation between ALBI grade and VRC *C*_trough_. Moreover, multivariate analysis identified ALBI grade as independent predictors of VRC exposure. This result has important clinical implications. The CP classification includes relatively subjective clinical assessments, such as ascites and hepatic encephalopathy. In patients with HIV-infected, these features may be confounded by other conditions. For example, systemic edema due to severe hypoalbuminemia, including ascites-like fluid accumulation, may be misattributed to hepatic ascites. Likewise, central nervous system infections or HIV-associated encephalopathy can be difficult to distinguish from hepatic encephalopathy. This lack of specificity may reduce the accuracy of the CP classification in reflecting intrinsic hepatic metabolic function.

In contrast, the ALBI grade is derived solely from two objective laboratory parameters: ALB and TBIL. ALB is a sensitive marker of hepatic synthetic function and may influence VRC pharmacokinetics through two mechanisms. First, hypoalbuminemia may indicate impaired hepatic synthetic capacity and, indirectly, reduced activity of drug-metabolizing enzymes such as CYP2C19 and CYP3A4 (Mu et al., 2025). Second, because ALB is the primary binding protein for VRC, lower albumin concentrations increase the unbound fraction of VRC in plasma (Vanstraelen et al., 2014a; Vanstraelen et al., 2014b). Although we measured total VRC concentrations, hypoalbuminemia may still correspond to a clinically meaningful increase in free drug exposure, particularly in patients with hepatic impairment. Elevated TBIL reflects impaired biliary excretion or hepatocellular injury and is likewise associated with reduced VRC clearance (Tang et al., 2021). By integrating these two parameters, the ALBI score may more accurately capture liver function changes relevant to VRC metabolism and elimination, which likely explains its superior predictive performance in our statistical analyses. Collectively, these findings suggest that, in patients with HIV-infected, the ALBI grade may provide a more reliable and practical approach than the CP classification for estimating VRC exposure and guiding initial dose selection.

This study also identified associations between inflammatory, nutritional, and immune status and VRC plasma concentrations, providing additional insight into the pharmacokinetic characteristics of this patient population. First, we observed a significant positive correlation between CRP and VRC *C*_trough_, with markedly higher trough levels in patients with CRP >100 mg/L. A plausible mechanism is that severe inflammation downregulates hepatic cytochrome P450 (CYP) enzyme expression and activity, particularly CYP2C19 and CYP3A4, through proinflammatory cytokines such as interleukin-6 (IL-6) (Aitken and Morgan, 2007). Because VRC is metabolized predominantly by CYP2C19 and, to a lesser extent, CYP3A4, systemic inflammation associated with severe infection may reduce metabolic clearance and thereby increase plasma concentrations. In patients with HIV-infected, profound immunosuppression and opportunistic infections frequently trigger intense, poorly controlled inflammatory responses, which may further amplify this inflammation–drug interaction (Shah and Smith, 2015).

In addition, patients with hypoalbuminemia exhibited higher VRC *C*_trough_. As noted previously, hypoalbuminemia may indicate impaired hepatic function and also reflects altered protein binding. Under conditions of low ALB levels, even when the total drug concentration falls within the therapeutic window, the unbound concentration may already exceed the safety threshold (Vanstraelen et al., 2014b). This phenomenon may partially explain why some patients with HIV-infected experience VRC-related toxicity despite having total concentrations within the therapeutic range. Free VRC concentrations were not measured in this study, precluding direct validation of this hypothesis. Nevertheless, this limitation identifies an important direction for future research.

Although a positive dose–concentration relationship would be expected under ideal conditions, we did not observe a significant correlation between VRC dose and *C*_trough_ (*P* = 0.605). This may be explained by the high interindividual pharmacokinetic variability (CV = 63.6%), TDM-driven dose adjustments during therapy, confounding by liver function (patients with hepatic impairment may receive lower doses but have higher concentrations), and the limited sample size. These factors collectively obscure the dose–concentration relationship in real-world clinical settings.

Multivariable analysis identified an independent inverse association between the CD4^+^/CD8^+^ ratio and VRC *C*_trough_. Although this observation is novel and warrants further investigation, its mechanistic basis cannot be established from the present dataset. One possibility is that the CD4^+^/CD8^+^ ratio reflects chronic immune dysregulation and susceptibility to opportunistic infections, which may predispose patients to an inflammatory milieu that suppresses drug-metabolizing enzymes and reduces VRC clearance (Obeagu and Obeagu, 2026; Garrido-Rodríguez et al., 2024). Notably, the CD4^+^/CD8^+^ ratio remained associated with *C*_trough_ after adjustment for CRP, suggesting that it captures aspects of host status not fully reflected by a single contemporaneous CRP measurement. An alternative hypothesis is a direct effect of HIV-related immune dysregulation on hepatic drug-metabolizing capacity (Jetter et al., 2010); however, evidence for this pathway is limited and it could not be evaluated in our study. We selected the CD4^+^/CD8^+^ ratio rather than absolute CD4^+^ count because CD4^+^ counts were markedly low in this cohort (median 55 cells/µL), resulting in limited discriminatory capacity at the lower end. Future prospective studies incorporating immune activation biomarkers (e.g., IL-6, TNF-α) and longitudinal sampling are needed to test whether immune activation mediates the observed association and to clarify its implications for individualized VRC dosing.

With respect to concomitant medications, concomitant corticosteroid use was significantly associated with lower VRC *C*_trough_. This finding is consistent with the CYP3A4-inducing properties of corticosteroids, which would be expected to decrease VRC exposure. Notably, a similar finding has been reported in other studies (Jia et al., 2021). The observed glucocorticoid-associated reduction in VRC *C*_trough_ (β = −2.34, approximately 50%) appears larger than what would be predicted by CYP3A4 induction alone, given the doses administered in our cohort (dexamethasone 5 mg/day, methylprednisolone 20–40 mg/day, hydrocortisone 300 mg/day). Although corticosteroids can induce CYP3A4, the magnitude of the observed reduction in *C*_trough_ may not be fully explained by CYP3A4 induction alone. Glucocorticoid-associated decreases in VRC exposure have been reported previously, and induction of additional pathways (e.g., CYP2C19) has been suggested (Dolton et al., 2012); however, our study did not assess CYP activity or genotype, and therefore mechanistic inference is not possible. Further studies are needed to elucidate this interaction. In contrast, PPIs were not significantly associated with VRC *C*_trough_ in our study. This may be attributed to the fact that no patients received omeprazole, a potent CYP2C19 inhibitor, and most patients instead received rabeprazole or pantoprazole, both of which have weaker inhibitory effects on CYP2C19. One patient received darunavir/cobicistat in combination with albuvirtide. Although this combination has the potential for drug-drug interaction with VRC via CYP3A4 inhibition, coadministration was considered permissible under TDM guidance. This patient’s VRC *C*_trough_ was 1.93 mg/L (within the therapeutic range), and dose adjustment was not required. Therefore, this patient was not excluded from the analysis.

The observed ADR incidence in our cohort was 20.5%. Although this rate may appear relatively high, it likely reflects the vulnerability of HIV-infected patients with IFD, who frequently experience polypharmacy, hepatic dysfunction, and profound immunosuppression. Sensitivity analyses excluding events classified as mild ADRs and those rated as “possible” on the Naranjo causality scale yielded similar optimal cutoffs, supporting the robustness of our primary findings to ADR case definition. Regarding safety, we identified a potential VRC *C*_trough_ threshold of 4.43 mg/L for predicting ADRs, which is lower than the commonly recommended upper limit of 5.5 mg/L. At this threshold, the sensitivity was 66.7% (one-third of ADR patients missed), specificity 80.4%, PPV 40.0%, and NPV 92.5%. The high NPV supports ruling out ADRs, while the moderate PPV reflects the multifactorial nature of VRC toxicity. However, this finding should be interpreted with caution due to the limited number of ADR events (n = 9) and the exploratory nature of the analysis. The suboptimal sensitivity may be explained by heterogeneity of ADR types, individual susceptibility, and variability in the timing of ADR onset. Nevertheless, our findings suggest that HIV-infected patients may be more vulnerable to VRC toxicity. We propose this cutoff as an alert threshold rather than a strict therapeutic target. Clinicians should remain vigilant for ADRs even below this cutoff, particularly in patients with advanced immunosuppression. Larger prospective studies are needed for validation.

In this study, CYP2C19 genotyping was not routinely performed in patients receiving VRC. This was primarily attributable to the high cost of genetic testing and the lack of established clinical genotyping infrastructure during the study period. Accordingly, our analyses focused on non-genetic determinants of VRC *C*_trough_ and did not account for pharmacogenetic factors such as CYP2C19 genotype. The observed associations between ALBI grade, corticosteroid use, CD4^+^/CD8^+^ ratio, and *C*_trough_ may therefore be partly confounded by unmeasured CYP2C19 variation, which could either amplify or attenuate the effects of these covariates on VRC exposure.

This limitation is particularly relevant in East Asian populations, including Han Chinese, in whom CYP2C19 loss-of-function alleles are more prevalent than in European populations; approximately 13%–23% of individuals are classified as poor metabolizers (PMs) (Takesue et al., 2022). PMs have been associated with markedly higher VRC exposure (approximately fourfold) (Weiss et al., 2009), which could contribute substantially to the interindividual variability observed in our cohort (CV, 63.6%). Conversely, the CYP2C19*17 allele, which is associated with rapid metabolism and potentially subtherapeutic concentrations, occurs at a lower but clinically meaningful frequency (∼2%–5%) in Chinese populations (Moriyama et al., 2017); failure to identify these individuals may increase the risk of underexposure and treatment failure. Emerging evidence also suggests that the magnitude of CYP2C19 genotype effects on steady-state VRC *C*_trough_ may vary by age in Chinese patients (Du et al., 2024).

Increasing data support the integration of CYP2C19 genotype-guided dosing with TDM as a precision-medicine approach that can improve target attainment and reduce toxicity. For example, Katada et al. (Katada et al., 2025) reported in a Japanese cohort that CYP2C19-guided VRC dosing reduced the composite incidence of adverse events (hepatotoxicity and visual disturbances) compared with standard dosing while maintaining efficacy. In our study, the relatively high ADR incidence (20.5%) may, at least in part, reflect the absence of genotype-informed dosing. Future work should incorporate pharmacogenomic testing into the TDM framework for VRC therapy in HIV-infected patients to further optimize dosing and improve safety.

## Limitations

This study has several limitations. First, the single-center retrospective design may introduce selection and information bias, and the relatively small sample size limits statistical power for subgroup analyses. Second, the lack of unbound VRC concentration measurements prevents a more direct evaluation of the mechanistic contribution of hypoalbuminemia. Third, CYP2C19 genotyping was not performed, despite its established role as a key genetic determinant of VRC pharmacokinetics. Future prospective, multicenter studies incorporating CYP2C19 genotyping and free drug concentration monitoring are warranted. Retrospective identification of ADRs is inherently susceptible to underreporting and documentation bias, particularly for mild or transient symptoms. Although the observed incidence (20.5%) falls within the range reported in previous studies (Chen et al., 2025; Kim et al., 2011), suggesting that substantial under-ascertainment is unlikely, prospective active surveillance remains the preferred approach for future investigations.

## Conclusion

This study systematically characterized the substantial interindividual variability in VRC *C*_trough_ among patients with HIV-infected complicated by IFD, underscoring the necessity of routine TDM in this population. VRC *C*_trough_ was independently associated with ALBI grade, concomitant glucocorticoid use, and the CD4^+^/CD8^+^ ratio. CRP showed a non-significant positive trend, and the univariate associations with hypoalbuminemia and CP class were attenuated after accounting for multicollinearity with ALBI grade. From a safety perspective, we identified an alert threshold of 4.43 mg/L for VRC-related ADRs in patients with HIV-infected, suggesting greater vulnerability to toxicity than that implied by the conventional therapeutic range and supporting a more conservative approach to concentration control. Future validation studies should incorporate pharmacogenomic profiling to refine individualized dosing strategies.

## Data Availability

The original contributions presented in the study are included in the article/Supplementary Material, further inquiries can be directed to the corresponding authors.
