# Supplementary material for: Voriconazole therapeutic drug monitoring and safety in HIV-infected patients with invasive fungal disease
Source: Front Pharmacol. 2026 Jun 19;17:1837833. doi: 10.3389/fphar.2026.1837833 (PMC13328384; doi:10.3389/fphar.2026.1837833)
Supplement: Supplementary file 1 [file Table1.docx]

**Supplementary table**

**Table 3. Specific ART regimens with corresponding** **VRC** ***C*_trough_ values**

| Patient ID | ART regimens | VRC *C*_trough_ values |
| --- | --- | --- |
| 1 | Bictegravir/Emtricitabine/Tenofovir Alafenamide + Albuvirtide | 2.44 |
| 2 | Bictegravir/Emtricitabine/Tenofovir Alafenamide + Albuvirtide | 0.88 |
| 3 | Bictegravir/Emtricitabine/Tenofovir Alafenamide | 4.84 |
| 4 | Bictegravir/Emtricitabine/Tenofovir Alafenamide | 3.07 |
| 5 | Lamivudine/Dolutegravir + Albuvirtide | 2.43 |
| 6 | NA | 5.20 |
| 7 | Bictegravir/Emtricitabine/Tenofovir Alafenamide + Albuvirtide | 5.43 |
| 8 | NA | 2.36 |
| 9 | Lamivudine/Dolutegravir | 3.57 |
| 10 | Lamivudine/Dolutegravir | 1.54 |
| 11 | Lamivudine/Dolutegravir | 5.38 |
| 12 | Lamivudine/Dolutegravir | 1.56 |
| 13 | Bictegravir/Emtricitabine/Tenofovir Alafenamide | 5.63 |
| 14 | Lamivudine/Dolutegravir | 5.86 |
| 15 | Bictegravir/Emtricitabine/Tenofovir Alafenamide | 5.96 |
| 16 | Bictegravir/Emtricitabine/Tenofovir Alafenamide | 3.15 |
| 16 | Bictegravir/Emtricitabine/Tenofovir Alafenamide | 3.73 |
| 17 | Lamivudine/Dolutegravir | 2.41 |
| 17 | Lamivudine/Dolutegravir | 1.07 |
| 18 | Lamivudine/Dolutegravir | 3.63 |
| 19 | Bictegravir/Emtricitabine/Tenofovir Alafenamide | 3.85 |
| 20 | Lamivudine/Dolutegravir | 0.07 |
| 20 | Lamivudine/Dolutegravir | 2.39 |
| 21 | NA | 5.79 |
| 21 | NA | 4.25 |
| 22 | NA | 4.42 |
| 23 | Lamivudine/Dolutegravir | 2.03 |
| 24 | Lamivudine/Dolutegravir | 0.20 |
| 25 | Lamivudine/Dolutegravir | 0.28 |
| 25 | Lamivudine/Dolutegravir | 1.52 |
| 25 | Lamivudine/Dolutegravir | 1.02 |
| 25 | Lamivudine/Dolutegravir | 1.57 |
| 26 | Bictegravir/Emtricitabine/Tenofovir Alafenamide | 3.19 |
| 27 | Bictegravir/Emtricitabine/Tenofovir Alafenamide | 5.15 |
| 28 | Bictegravir/Emtricitabine/Tenofovir Alafenamide + Albuvirtide | 0.14 |
| 28 | Bictegravir/Emtricitabine/Tenofovir Alafenamide + Albuvirtide | 1.85 |
| 29 | Lamivudine/Dolutegravir | 3.09 |
| 30 | Lamivudine/Dolutegravir | 0.42 |
| 30 | Lamivudine/Dolutegravir | 1.67 |
| 31 | Bictegravir/Emtricitabine/Tenofovir Alafenamide | 5.69 |
| 32 | Bictegravir/Emtricitabine/Tenofovir Alafenamide | 2.34 |
| 33 | Bictegravir/Emtricitabine/Tenofovir Alafenamide + Albuvirtide | 5.93 |
| 33 | Bictegravir/Emtricitabine/Tenofovir Alafenamide + Albuvirtide | 5.47 |
| 34 | Lamivudine/Dolutegravir + Albuvirtide | 0.321 |
| 35 | Bictegravir/Emtricitabine/Tenofovir Alafenamide | 5.88 |
| 36 | Darunavir/Cobicistat + Albuvirtide | 1.93 |
| 37 | Bictegravir/Emtricitabine/Tenofovir Alafenamide | 7.26 |
| 37 | Bictegravir/Emtricitabine/Tenofovir Alafenamide | 1.04 |
| 38 | Lamivudine/Dolutegravir | 0.89 |
| 39 | Lamivudine/Dolutegravir | 4.44 |
| 40 | Lamivudine/Dolutegravir | 3.50 |
| 41 | Lamivudine/Dolutegravir | 1.18 |
| 42 | Lamivudine/Dolutegravir | 2.68 |
| 43 | Lamivudine/Dolutegravir | 1.66 |
| 44 | NA | 1.43 |
