# Supplementary material for: Voriconazole therapeutic drug monitoring and safety in HIV-infected patients with invasive fungal disease
Source: Front Pharmacol. 2026 Jun 19;17:1837833. doi: 10.3389/fphar.2026.1837833 (PMC13328384; doi:10.3389/fphar.2026.1837833)
Supplement: Supplementary file 2 [file Table2.docx]

**Supplementary table**

**Table 4. The individual VRC *C*_trough_ values for the nine ADR cases**

| Patient ID | VRC *C*_trough_ values |
| --- | --- |
| 1 | 0.88 |
| 2 | 2.43 |
| 3 | 5.63 |
| 4 | 5.79 |
| 5 | 2.03 |
| 6 | 5.15 |
| 7 | 5.93 |
| 8 | 5.88 |
| 9 | 4.44 |
